# Elp1 function in placode‐derived neurons is critical for proper trigeminal ganglion development

**DOI:** 10.1002/dvdy.749

**Published:** 2024-10-09

**Authors:** Margaret A. Hines, Lisa A. Taneyhill

**Affiliations:** ^1^ Department of Animal and Avian Sciences University of Maryland College Park Maryland USA

**Keywords:** Elp1, neural crest cells, placode‐derived neurons, trigeminal ganglion

## Abstract

**Background:**

The trigeminal nerve is the largest cranial nerve and functions in somatosensation. Cell bodies of this nerve are positioned in the trigeminal ganglion, which arises from the coalescence of neural crest and placode cells. While this dual cellular origin has been known for decades, the molecular mechanisms controlling trigeminal ganglion development remain obscure. We performed RNA sequencing on the forming chick trigeminal ganglion and identified *Elongator acetyltransferase complex subunit 1* (*Elp1*) for further study. Mutations in *ELP1* cause familial dysautonomia (FD), a fatal disorder characterized by the presence of smaller trigeminal nerves and sensory deficits. While Elp1 has established roles in neurogenesis, its function in placode cells during trigeminal gangliogenesis has not been investigated.

**Results:**

To this end, we used morpholinos to deplete Elp1 from chick trigeminal placode cells. Elp1 knockdown decreased trigeminal ganglion size and led to aberrant innervation of the eye by placode‐derived neurons. Trigeminal nerve branches also appeared to exhibit reduced axon outgrowth to target tissues.

**Conclusions:**

These findings reveal a new role for Elp1 in placode‐derived neurons during chick trigeminal ganglion development. These results have potential high significance to provide new insights into trigeminal ganglion development and the etiology of FD.

## Key Findings

- Elp1 is expressed in undifferentiated neural crest cells and placode‐derived neurons contributing to the trigeminal ganglion.
- Elp1 knockdown in trigeminal placode cells reduces trigeminal ganglion size.
- Elp1 depletion from trigeminal placode cells leads to aberrant target tissue innervation.

## 1 INTRODUCTION

Cranial sensory nerves are components of the peripheral nervous system that are responsible for relaying sensory information to the central nervous system. The structure that houses the neuronal cell bodies and supporting glia of the sensory nerves is termed the ganglion. In the head, these include the trigeminal (V) and epibranchial (geniculate (facial VII), petrosal (glossopharyngeal IX), and nodose (vagal X)) ganglia. Nerves arising from these ganglia innervate diverse structures including the face, tongue, mouth, and digestive tract, and are derived from two embryonic cell populations, cranial neural crest and neurogenic placodes.

The trigeminal ganglion and its associated nerves are responsible for detecting pain, touch, and temperature sensations in the head and face.^1^ Neural crest cells from the midbrain and rostral hindbrain regions, along with placode cells from the trigeminal placode, migrate and coalesce together to form this ganglion. While often referred to as one structure, the trigeminal placode is made up of two distinct placodes, the ophthalmic and maxillomandibular,^2^, ^3^ each contributing to a specific lobe of the trigeminal ganglion. Unlike other cranial sensory ganglia, neurons derived from neural crest cells and placode cells are intermixed throughout this ganglion.^3^, ^4^ Reciprocal interactions between these cell types are required for proper trigeminal ganglion formation^3^, ^5^, ^6^, ^7^; however, the molecules and pathways mediating ganglion development remain elusive.

Through RNA sequencing of wildtype embryonic trigeminal ganglia,^8^ we identified *Elongator acetyltransferase complex subunit 1* (*Elp1*) as a potential candidate for controlling chick trigeminal ganglion development. Elp1 was originally discovered as the scaffolding subunit of the six‐subunit dimeric Elongator complex, which regulates transcriptional elongation through interactions with RNA polymerase II.^9^, ^10^ However, numerous roles for Elongator have been discovered in other contexts, including the regulation of translation through tRNA modifications, kinase signaling, actin organization, exocytosis, and acetylation of alpha‐tubulin and synaptic proteins.^9^ Further, many of these functions are vital for neurodevelopment, as they aid in migration, neuritogenesis, and trafficking. Intriguingly, mutations in different Elongator complex subunits give distinct phenotypes and cause, or are associated with, specific human diseases and conditions,^9^ supporting the notion of Elongator‐dependent and ‐independent roles for Elp subunits. For example, mutations in *ELP1* cause familial dysautonomia (FD), a neurodevelopmental and neurodegenerative disease.^11^ Evidence such as smaller trigeminal nerves in patients (and in FD mouse models) and symptoms such as decreased sensitivity to pain and temperature in the face, absent corneal reflexes, and reduced basal lacrimation, suggest the presence of trigeminal nerve deficits in FD.^12^, ^13^, ^14^, ^15^ A recent report examining the loss of Elp1 in neural crest‐derived neurons of the mouse trigeminal ganglion confirms this, revealing axon outgrowth and target tissue innervation defects.^16^

However, much of our insight on Elp1 function comes from studies on neural crest‐derived neurons in the trunk, either using conditional knockout mouse models in which *Elp1* is deleted from neural crest cells and their derivatives through Wnt1‐Cre‐mediated recombination^13^, ^17^, ^18^ or via knockdown methods in the chick embryo.^19^, ^20^ Examination of trunk neural crest‐derived sensory ganglia in these mouse models revealed that Elp1 deletion caused second wave neuronal progenitors to differentiate early or undergo cell death, leading to fewer neurons and thus smaller ganglia.^17^ Depletion of Elp1 in the chick led to precocious neuronal differentiation, increased axon branching, and premature cell death in one study while another identified abnormal axon branching, including an increase in branching points, as well as neuron guidance abnormalities and defects in axonal transport.^19^, ^20^ These results add to the data from mouse models and strongly suggest roles for Elp1 in nerve outgrowth and/or target tissue innervation, neuron survival, and protein trafficking.^13^, ^17^, ^18^, ^21^, ^22^

Given its dual cellular origin, understanding the full function of Elp1 in the trigeminal ganglion requires investigating the role of Elp1 in placode‐derived neurons as well. Since there is currently no Cre driver that only targets trigeminal placodes, we turned to the chick embryo. We first characterized Elp1 spatio‐temporal distribution as the trigeminal ganglion initially assembles from undifferentiated neural crest cells and placode‐derived neurons. Our data show that Elp1 is expressed in migratory cranial neural crest cells and later in undifferentiated neural crest cells and placode‐derived neurons contributing to the trigeminal ganglion. Next, we performed morpholino‐mediated knockdown of Elp1 in trigeminal placode cells and uncovered a sustained negative impact on trigeminal ganglion development. Our results are the first to describe Elp1 expression in the forming chick trigeminal ganglion and point to critical functions for Elp1 in placode‐derived neurons during trigeminal ganglion development, providing additional insight into the etiology of trigeminal nerve deficits in FD.

## 2 RESULTS

### 2.1 Elp1 is dynamically expressed throughout trigeminal ganglion development

**FIGURE 1:**
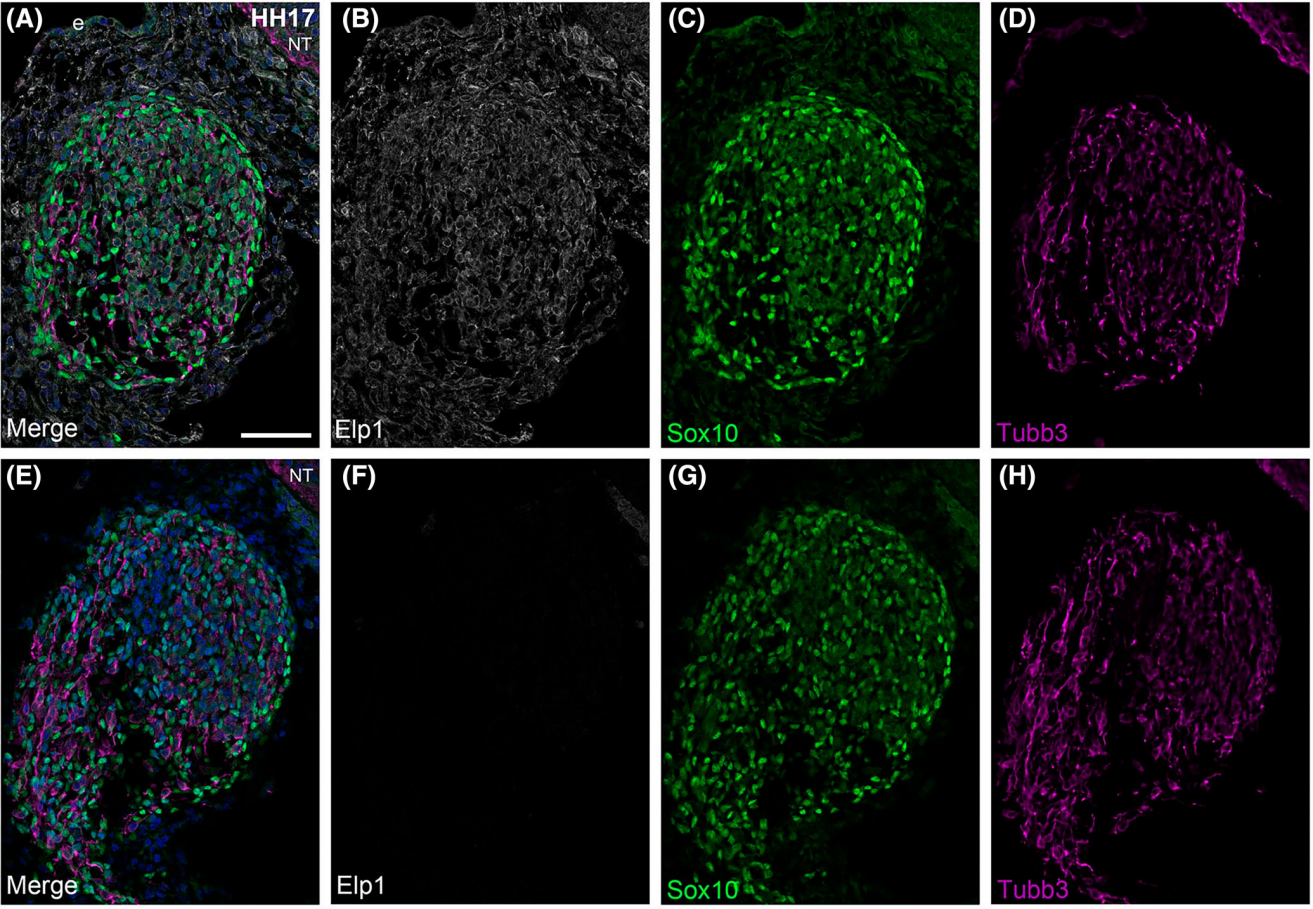
Secondary antibody‐only control for Elp1 immunohistochemistry supports the specificity of the Elp1 antibody. Representative transverse section through the forming trigeminal ganglion ophthalmic lobe (E2.5/HH17, *n* = 3) followed by immunohistochemistry for Elp1 (A,B, white), Sox10 (A,C,E,G, green, labels neural crest cells), and Tubb3 (A,D,E,H, purple, labels placode‐derived neurons) with corresponding merged images of all channels with DAPI (A,E, blue, marks all nuclei). Secondary antibody‐only control with absence of a primary antibody for Elp1 yields no detectable Elp1 fluorescence (E,F). The scale bar in (A) is 50 μm and applies to all images. e = ectoderm; NT = neural tube.

Characterization of Elp1 protein expression was performed in stages, with embryos grouped every 12–24 hours, as this correlates with the timeframe of major developmental events (e.g., neural crest cell migration, initial intermixing of neural crest cells and placode‐derived neurons, and condensation of neural crest cells and placode‐derived neurons). A control experiment was first performed to ascertain the specificity of the Elp1 antibody (Figure 1). Immunostaining for Elp1 (Figure 1A,B) was performed in combination with Sox10 (Figure 1A,C) and Tubb3 (Figure 1A,D) to identify neural crest cells and placode‐derived neurons of the trigeminal ganglion, respectively.^23^ Next, immunostaining was conducted in the absence of Elp1 antibody but in the presence of a secondary antibody specific for Elp1 (Figure 1E,F), accompanied by the same combination of Sox10 (Figure 1E,G) and Tubb3 (Figure 1E,H) antibodies. This latter experiment showed no signal for Elp1, confirming the specificity of the Elp1 antibody, which was then used for subsequent characterization of the spatiotemporal expression pattern of Elp1.

**FIGURE 2:**
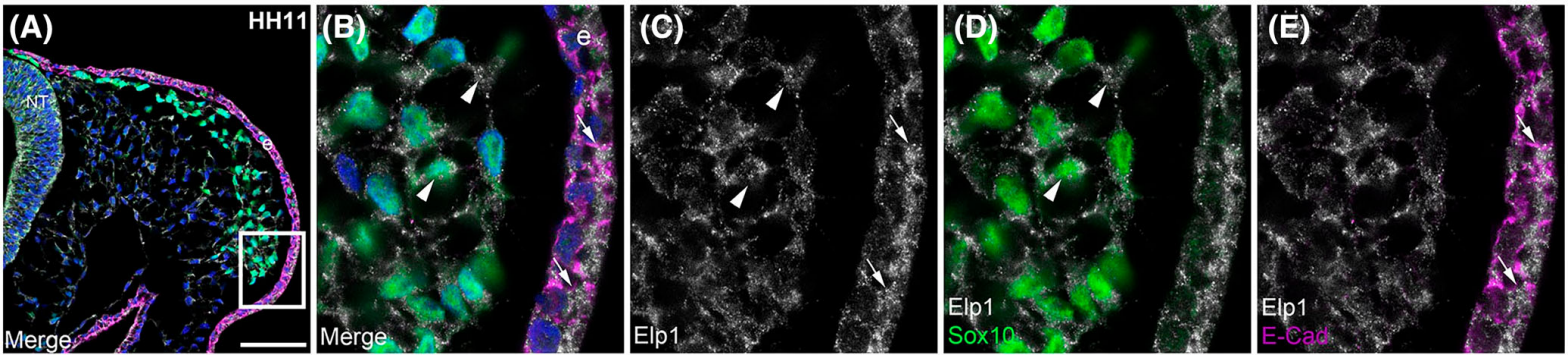
Elp1 is expressed in migratory neural crest cells and the surface ectoderm containing trigeminal placode cells prior to trigeminal ganglion assembly. Representative transverse section through an E1.5/HH11 (*n* = 3) embryo midbrain followed by immunohistochemistry for Elp1 (A–E, white), Sox10 (A,B,D, green, labels migratory neural crest cells), and E‐cadherin (E‐Cad, A,B,E, purple, labels the ectoderm), with corresponding merged images of all channels with DAPI (A,B, blue, marks all nuclei). A higher magnification image of the boxed region in (A) is shown in (B–E), with merge images of Elp1 and Sox10 (D, white and green, respectively) and Elp1 and E‐Cad (E, white and purple, respectively). Arrowheads indicate Elp1 in migratory neural crest cells (B–D), while arrows point to Elp1 in the ectoderm (B,C,E). Scale bar in (A) is 50 μm and applies to all images but is 10 μm for (B–E). NT = neural tube; e = ectoderm.

The first stage group investigated was embryonic day (E) 1.5/Hamburger–Hamilton (HH)11/12, when neural crest cells are migrating away from the dorsal neural tube to their respective locations. At these timepoints, placode cells reside in the ectoderm and have yet to start their delamination and migration. Within Sox10‐positive migratory neural crest cells, Elp1 expression is punctate in appearance (Figure 2B–D, arrowheads). Furthermore, Elp1 is observed in the ectoderm containing placodal precursors, demonstrated by its presence in E‐cadherin‐positive cells. This ectodermal expression appeared to be concentrated at the apical side of the cells (Figure 2B,C,E, arrows).

**FIGURE 3:**
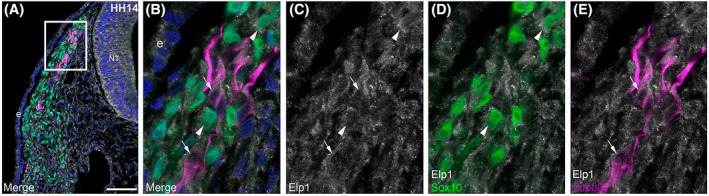
Elp1 is expressed in neural crest cells and placode‐derived neurons of the trigeminal ganglion during early placode cell delamination and migration. Representative transverse section through the forming trigeminal ganglion ophthalmic lobe (E2/HH14, *n* = 3) followed by immunohistochemistry for Elp1 (A–E, white), Sox10 (A,B,D, green, labels neural crest cells), and Tubb3 (A,B,E, purple, labels placode‐derived neurons), with corresponding merged images of all channels with DAPI (A,B, blue, marks all nuclei). A higher magnification image of the boxed region in (A) is shown in (B–E), with merge images of Elp1 and Sox10 (D, white and green, respectively) and Elp1 and Tubb3 (E, white and purple, respectively). Arrowheads denote Elp1 in migratory neural crest cells (B–D), while arrows indicate Elp1 in placode‐derived neurons (B,C,E). The scale bar in (A) is 50 μm and applies to all images but is 10 μm for (B–E). e = ectoderm.

At E2/HH13‐15, placode cells are delaminating from the ectoderm and differentiating into neurons, identified by their immunoreactivity for Tubb3 (Figure 3A,B,E). While Tubb3 eventually labels all neurons in the trigeminal ganglion, these neurons are placode‐derived at all stages in this expression profile, since neural crest cells differentiate much later, after E4.^24^ Elp1 expression (Figure 3A–E) is still present in the ectoderm (Figure 3C); however, the apical distribution of Elp1 has changed, with enrichment reduced compared to the previous stage group. We observed punctate expression of Elp1 in the cytoplasm and some nuclei (Figure 3B–D, arrowheads) of Sox10‐positive neural crest cells. Additionally, at these stages, Elp1 expression is noted in the cell bodies of Tubb3‐positive neurons (Figure 3B,C,E, arrows).

**FIGURE 4:**
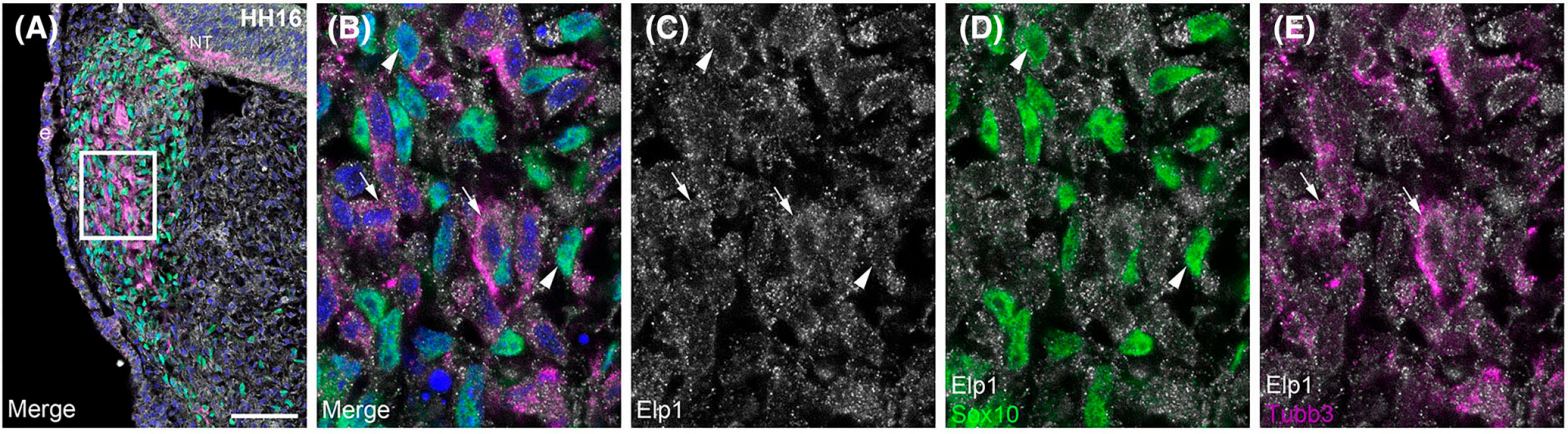
Elp1 is expressed in neural crest cells and placode‐derived neurons of the trigeminal ganglion during initial coalescence and condensation. Representative transverse section through the forming trigeminal ganglion ophthalmic lobe (E2.5/HH16, *n* = 3) followed by immunohistochemistry for Elp1 (A–E, white), Sox10 (A,B,D, green, labels neural crest cells), and Tubb3 (A,B,E, purple, labels placode‐derived neurons), with corresponding merged images of all channels with DAPI (A,B, blue, marks all nuclei). A higher magnification image of the boxed region in (A) is shown in (B–E), with merge images of Elp1 and Sox10 (D, white and green, respectively) and Elp1 and Tubb3 (E, white and purple, respectively). Arrowheads point to Elp1 in migratory neural crest cells (B–D), while arrows denote Elp1 in placode‐derived neurons (B,C,E). The scale bar in (A) is 50 μm and applies to all images but is 10 μm for (B–E). e = ectoderm; NT = neural tube.

**FIGURE 5:**
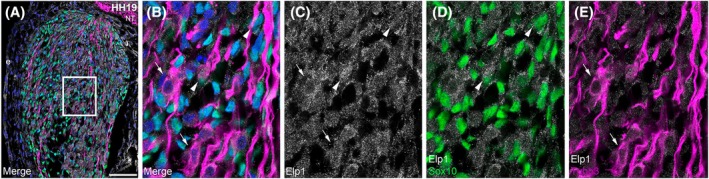
Elp1 expression persists in neural crest cells and placode‐derived neurons of the trigeminal ganglion at later stages. Representative transverse section through the forming trigeminal ganglion ophthalmic lobe (E3/HH19, *n* = 3) followed by immunohistochemistry for Elp1 (A–E, white), Sox10 (A,B,D green, labels neural crest cells), and Tubb3 (A,B,E, purple, labels placode‐derived neurons), with corresponding merged images of all channels with DAPI (A,B, blue, marks all nuclei). A higher magnification image of the boxed region in (A) is shown in (B–E), with merge images of Elp1 and Sox10 (D, white and green, respectively) and Elp1 and Tubb3 (E, white and purple, respectively). Arrowheads indicate Elp1 in migratory neural crest cells (B–D), while arrows point to Elp1 in placode‐derived neurons (B,C,E). The scale bar in (A) is 50 μm and applies to all images but is 10 μm for (B–E). e = ectoderm.

At E2.5/HH16‐17, neural crest cells and placode‐derived neurons have coalesced to form the trigeminal ganglion proper such that a “teardrop” or “semilunar” shape is observed in sections. Expression of Elp1 in Sox10‐positive neural crest cells (Figure 4B–D, arrowheads) and in the cell bodies of Tubb3‐positive neurons (Figure 4B,C,E, arrows) remains the same as in the previous stage grouping. Lastly, during E2.75–3.5/HH18‐20, a defined trigeminal ganglion structure is present, with more placode‐derived neurons surrounded by undifferentiated neural crest cells. Elp1 expression (Figure 5A–E) remains the same as observed in the previous stage groups, with ectodermal (Figure 5A), neural crest cell (Figure 5B–D, arrowheads), and neuronal (Figure 5B,C,E, arrows) expression noted. Taken together, these results indicate that Elp1 is expressed in the proper cell types and at the correct time to function in trigeminal ganglion development.

### 2.2 Elp1 morpholinos are effective at reducing Elp1 protein levels in trigeminal placode cells

**FIGURE 6:**
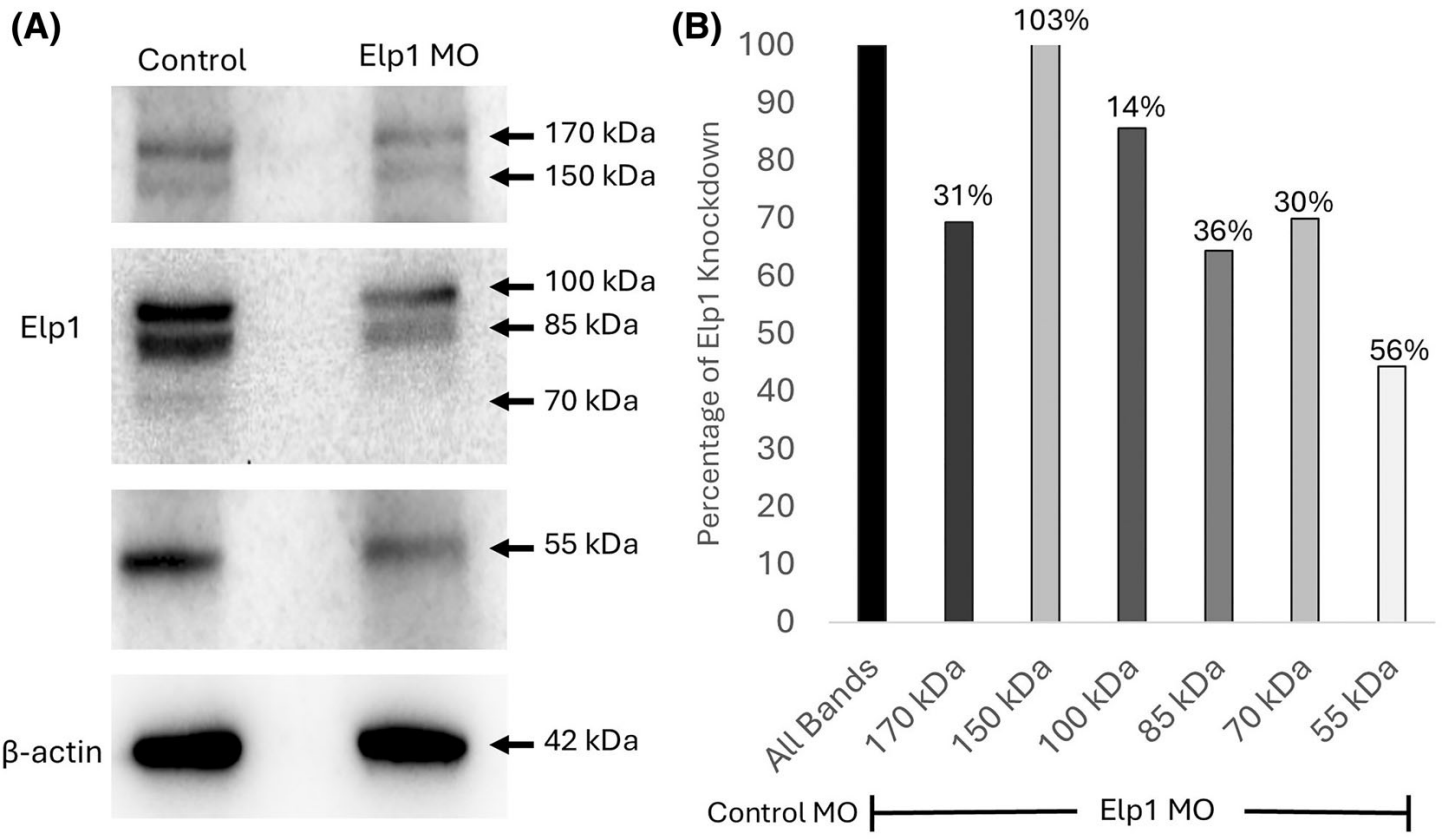
Elp1 MO reduces Elp1 protein levels in the trigeminal ganglion. The knockdown efficiency of a 1:1 ratio of electroporated *Elp1* translation‐blocking and *Elp1* splice‐blocking MO was assessed using immunoblotting on whole‐cell lysates prepared from dissected Elp1 MO‐ or Control MO‐positive trigeminal ganglia. (A). Six bands were immunoreactive to the Elp1 antibody and when normalized to β‐actin, five showed reduced intensities in the Elp1 MO‐treated tissue compared to the Control MO‐treated tissue (B).

A 1:1 mixture of an *Elp1* translation‐blocking morpholino and *Elp1* splice‐blocking morpholino (referred to hereafter as Elp1 MO), or a standard Control MO (GeneTools, LLC), was electroporated at E1/HH10‐11 using a unilateral ectodermal electroporation method^25^, ^26^ to target trigeminal placode cells prior to delamination. Immunoblotting was then performed with an Elp1 antibody previously validated for this method.^21^ This allowed us to determine the presence and size of Elp1 protein (i.e., bands) immunoreactive with this antibody in the trigeminal ganglion, since this has not been reported, and measure the level of Elp1 reduction to confirm efficacy of the Elp1 MO. Immunoblotting revealed six bands for Elp1, at molecular weights consistent with what has been reported previously for this antibody, five of which showed varying levels of knockdown ranging from 14% to 56% (Figure 6). The band at 150 kDa, however, showed no reduction in intensity after introduction of the Elp1 MO. These findings indicate that this combination of Elp1 MO can deplete Elp1 protein levels, providing a means by which to evaluate Elp1 function in the trigeminal ganglion.

### 2.3 Elp1 knockdown in trigeminal placode cells decreases the area of the trigeminal ganglion during the early stages of trigeminal ganglion assembly

**FIGURE 7:**
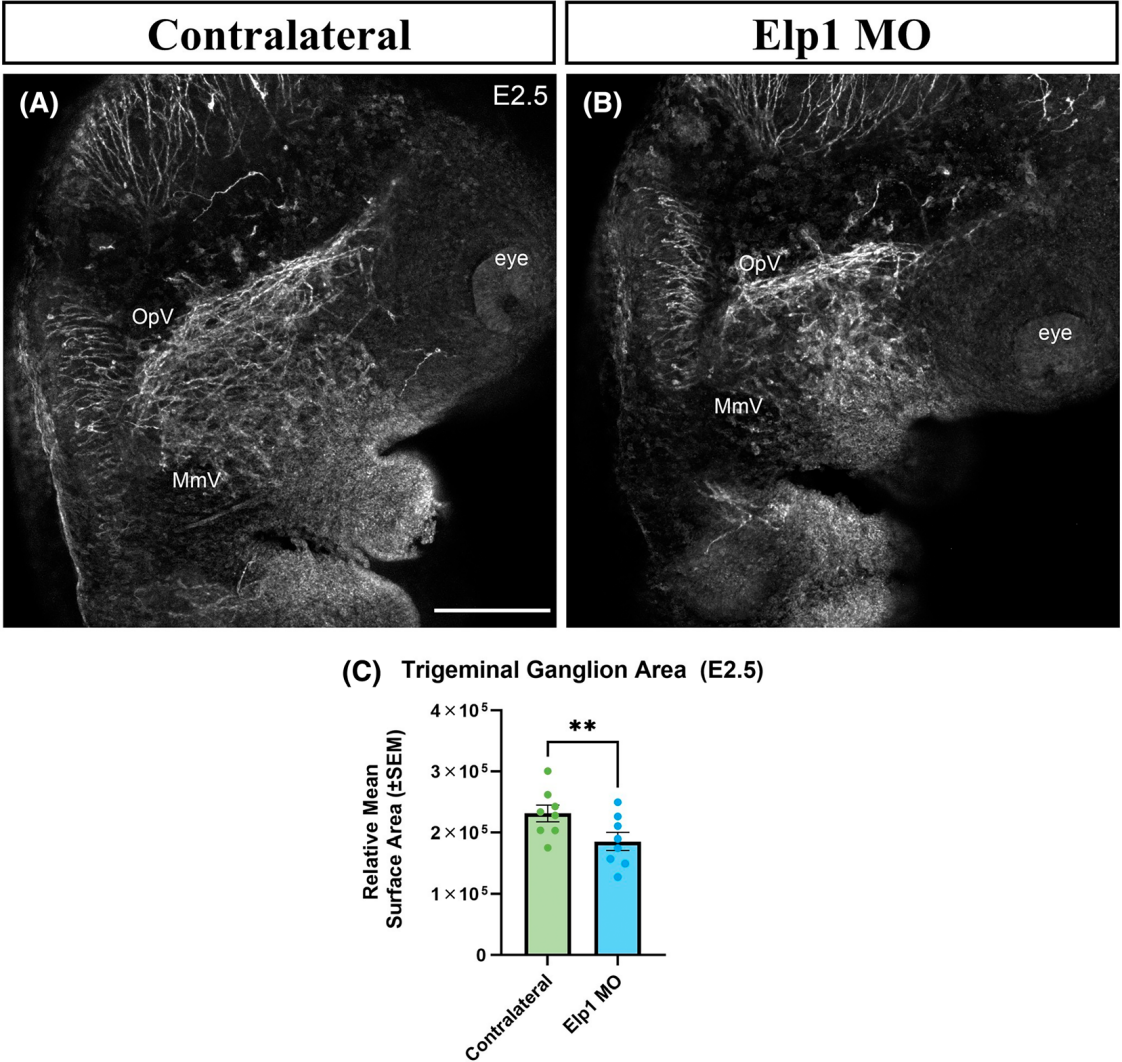
Reduction of Elp1 in trigeminal placode cells decreases trigeminal ganglion area. Representative max intensity projections of confocal Z‐stacks showing the contralateral control (A) or Elp1 MO‐treated (B) trigeminal ganglion at E2.5/HH16 after Tubb3 whole‐mount immunohistochemistry (white). Scale bar in (A) is 250 μm and applies to (B). (C) Quantification of the area of contralateral (green, *n* = 7) and Elp1 MO‐treated (blue, *n* = 7) trigeminal ganglia demonstrating statistical significance (*p* = .0014, paired *t*‐test). OpV = ophthalmic lobe; MmV = maxillomandibular lobe.

**FIGURE 8:**
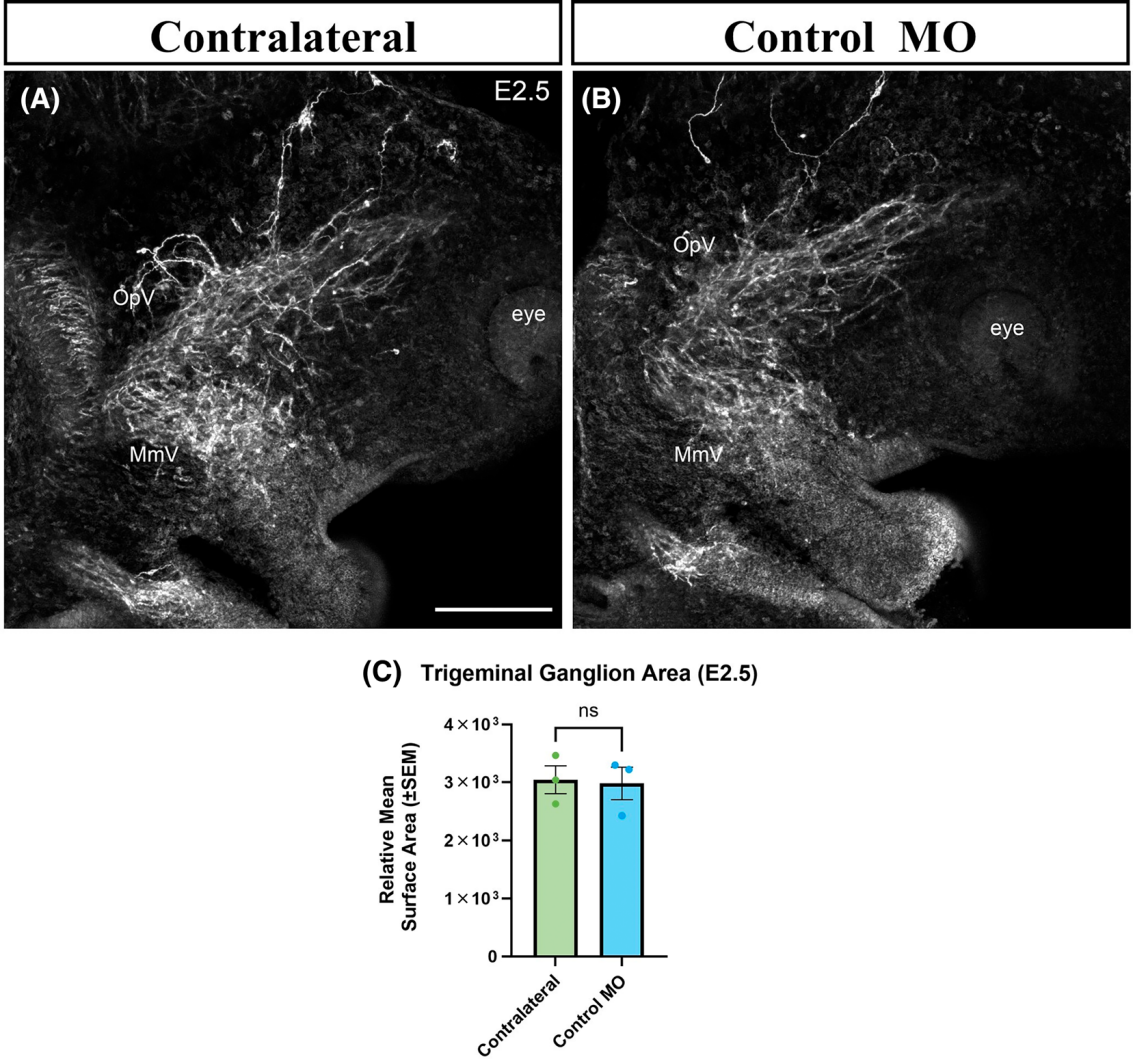
Electroporation of the scrambled Control MO into trigeminal placode cells does not alter trigeminal ganglion development. Representative max intensity projections of confocal Z‐stacks showing the Control MO‐treated (A) or contralateral control (B) trigeminal ganglion at E2.5/HH16 (*n* = 3) after Tubb3 whole‐mount immunohistochemistry (white). Scale bar in (A) is 250 μm and applies to (B). (C) Quantification of the area of contralateral (green, *n* = 3) and Control MO‐treated (blue, *n* = 3) trigeminal ganglia demonstrating no statistical significance (*p* = .734, paired *t*‐test). OpV = ophthalmic lobe; MmV = maxillomandibular lobe.

To investigate a role for Elp1 in trigeminal ganglion development, Elp1 MO was electroporated into trigeminal placode cells as described above. Trigeminal ganglion development was first evaluated at ~E2.5/HH15‐17, when placode‐derived neurons and migratory neural crest cells are intermingling, through whole‐mount immunohistochemical staining for Tubb3 to label placode‐derived neurons. Strikingly, defects were already apparent at these early stages of trigeminal ganglion development (Figure 7). On the contralateral (control) side of the embryo, the trigeminal ganglion formed normally, with ophthalmic and maxillomandibular lobes starting to become defined (Figure 7A). On the Elp1 MO‐treated side, knockdown of Elp1 negatively impacted trigeminal ganglion development (Figure 7B). Measurement of the area occupied by experimental and contralateral control side trigeminal ganglia revealed a statistically significant 20% decrease upon Elp1 knockdown compared to control (Figure 7C, *p* = .0014). Importantly, electroporation of a scrambled Control MO into trigeminal placode cells led to no phenotypic effect on trigeminal ganglion development (Figure 8), with results comparable to those observed on the contralateral control sides of both Elp1 MO‐ and Control MO‐treated embryos, and with no significant change in the area of the trigeminal ganglion (Figure 8C, *p* = .734). Therefore, the contralateral (nonelectroporated) sides of Elp1 MO‐treated embryos were used as controls in subsequent experiments to account for any developmental variation. Altogether, these data reveal early effects on trigeminal ganglion development upon knockdown of Elp1 in placode cells.

### 2.4 Elp1 knockdown in trigeminal placode cells causes persistent reduction in trigeminal ganglion area and negatively affects innervation of the eye

**FIGURE 9:**
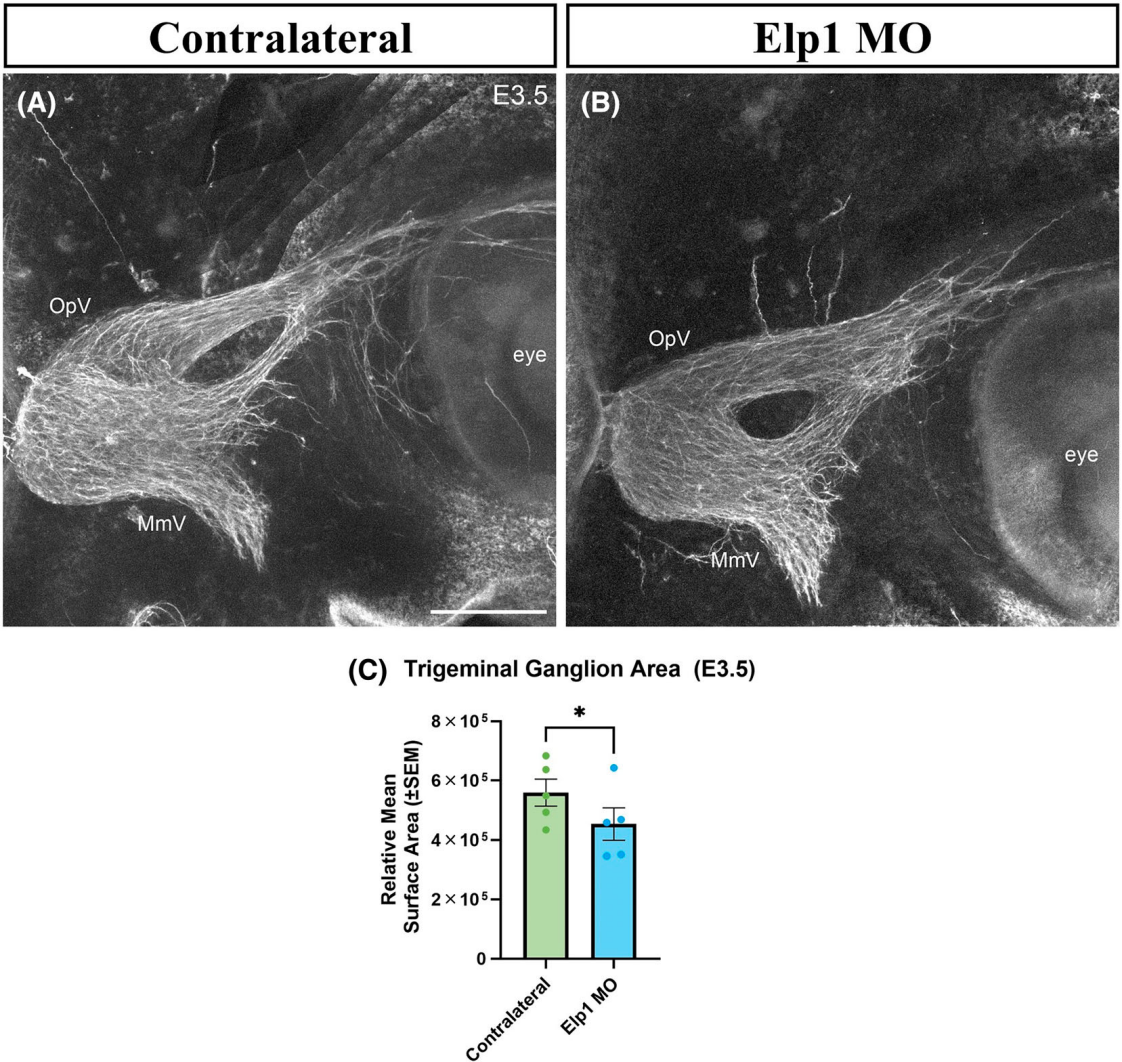
Elp1 depletion reduces the area of the trigeminal ganglion at later developmental stages. Representative max intensity projections of confocal Z‐stacks showing the contralateral control (A) or Elp1 MO‐treated (B) trigeminal ganglion at E3.5/HH19‐20 after Tubb3 whole‐mount immunohistochemistry (white). The scale bar in (A) is 250 μm and applies to (B). (C) Quantification of the area of contralateral (green, *n* = 7) and Elp1 MO‐treated (blue, *n* = 7) trigeminal ganglia demonstrating statistical significance (*p* = .0425, paired *t*‐test). OpV = ophthalmic lobe; MmV = maxillomandibular lobe.

One day later, extensive development of the trigeminal ganglion has occurred through further condensation of undifferentiated neural crest cells and placode‐derived neurons (E3.5/HH19‐20). Immunohistochemical staining for Tubb3 (marking placode‐derived neurons) revealed distinct ophthalmic, maxillary, and mandibular branches associated with the contralateral control trigeminal ganglion (Figure 9A). After Elp1 knockdown in trigeminal placode cells (Figure 9B), the area encompassed by the trigeminal ganglion remained significantly reduced, with a 20% decrease observed (Figure 9C, *p* = .0425). However, analysis of the widths for each branch uncovered no significant changes (data not shown).

**FIGURE 10:**
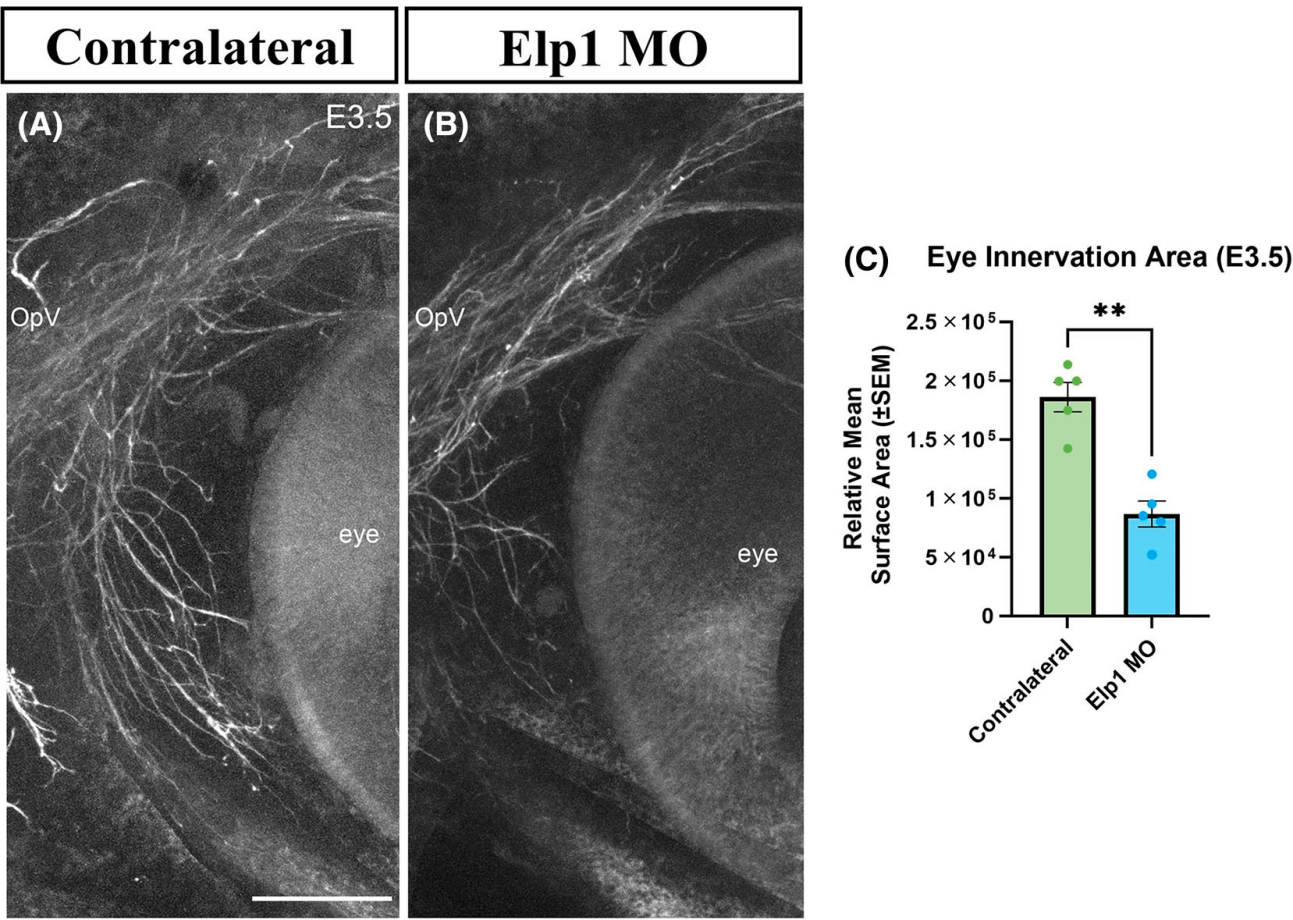
Reduction of eye innervation is observed after Elp1 knockdown. Representative max intensity projections of confocal Z‐stacks showing the contralateral control (A) or Elp1 MO‐treated (B) trigeminal ganglion at E3.5/HH19‐20 after Tubb3 whole‐mount immunohistochemistry (white) with a focus on the innervation of the eye by the ophthalmic branch. The scale bar in (A) is 300 μm and applies to (B). (C) Quantification of the area of contralateral (green, *n* = 5) and Elp1 MO‐treated (blue, *n* = 5) ophthalmic innervation of the eye demonstrating statistical significance (*p* = .004, paired *t*‐test). OpV = ophthalmic lobe.

Further examination of the ophthalmic branch innervation pattern showed normal axonal branching and projections targeting the contralateral control eye (Figure 10A). Innervation of the Elp1 MO‐treated side eye, however, was abnormal compared to the contralateral control side (Figure 10B). Quantification of this region (i.e., area occupied by ophthalmic axons) revealed a statistically significant reduction in the innervation of the eye after Elp1 knockdown (Figure 10C, 53%, *p* = .004) compared to the contralateral control side. Collectively, these data point to potential long‐term defects in the outgrowth of axons from placode‐derived neurons after Elp1 knockdown.

### 2.5 Elp1 knockdown in trigeminal placode negatively impacts placodal neurons

**FIGURE 11:**
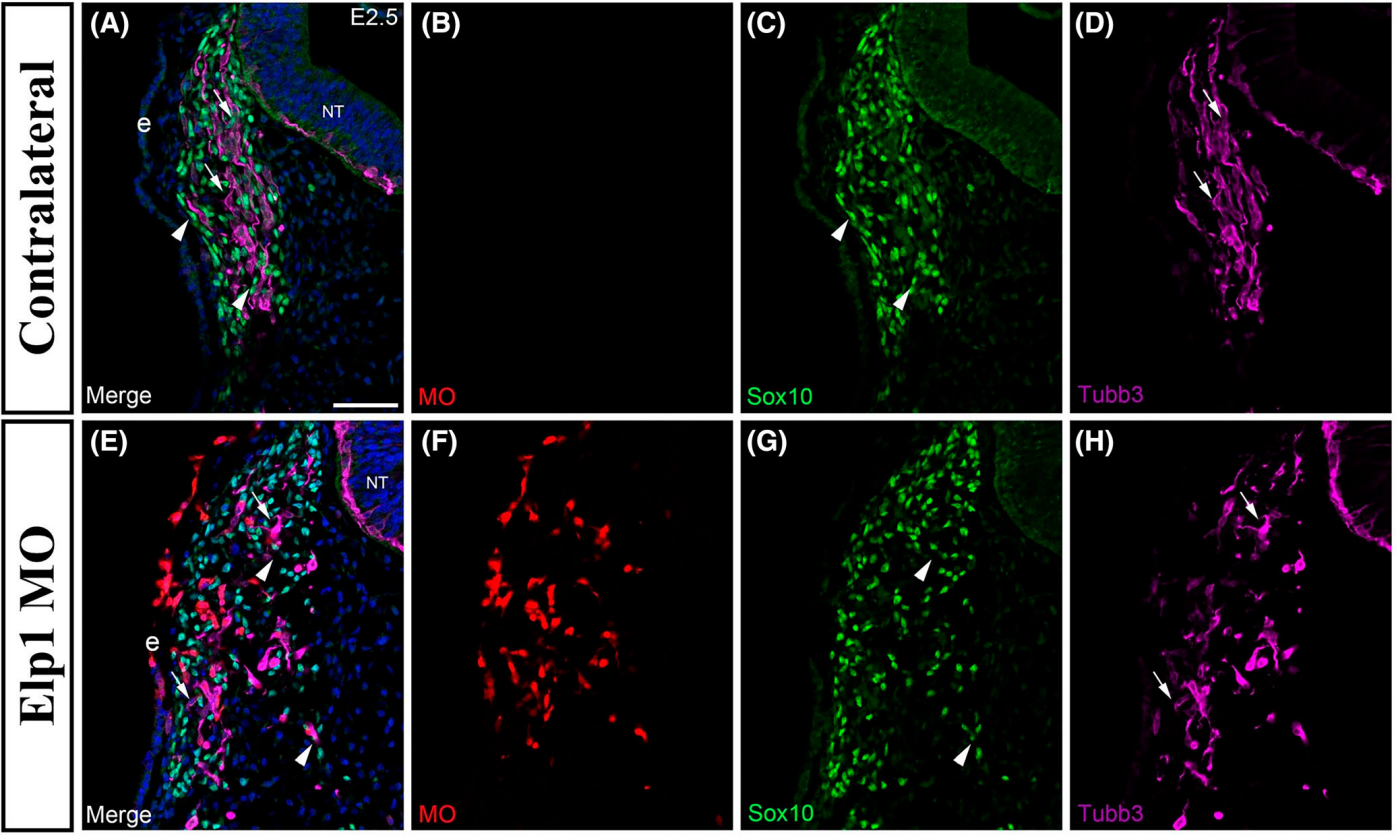
Elp1 knockdown impacts cell condensation within the trigeminal ganglion. Representative transverse section through the forming ophthalmic lobe of the trigeminal ganglion of an Elp1 MO‐treated embryo at E2.5/HH16, with the contralateral control and Elp1 MO‐treated sides shown in (A–D) and (E–H), respectively. Immunohistochemistry for Sox10 (C,G, green, labels neural crest cells) and Tubb3 (D,H, purple, labels placode‐derived neurons) was conducted on tissue sections. MO‐positive cells are visualized by the lissamine tag on the MO, which fluoresces red (F; none in B), with corresponding merged images of all channels with DAPI (A,E, blue, marks all nuclei). Arrowheads denote Sox10‐positive neural crest cells (A,C,E,G), while arrows point to Tubb3‐positive placode‐derived neurons (A,D,E,H). Scale bar in (A) is 50 μm and applies to all images. NT = neural tube; e = ectoderm.

To better discern potential changes at the cellular level after Elp1 knockdown, section immunohistochemistry was performed to identify placode‐derived neurons and undifferentiated neural crest cells. At early stages of development (E2.5/HH15‐17), the contralateral control trigeminal ganglion (Figure 11A–D) possessed the typical organization of Sox10‐positive neural crest cells (Figure 11A,C, arrowheads) surrounding Tubb3‐positive placode‐derived neurons (Figure 11A,D, arrows). Evaluation of the Elp1 MO‐treated trigeminal ganglion (Figure 11E–H), however, uncovered a change in the arrangement of neural crest cells and placode‐derived neurons after Elp1 knockdown. Condensation of placode‐derived neurons was aberrant, with Tubb3‐immunoreactive cells appearing less compact compared to the contralateral control trigeminal ganglion (Figure 11E,H, arrows). Furthermore, while neural crest cells still surrounded placode‐derived neurons, their distribution was now more spread out given the anatomical position of the placode‐derived neurons in the Elp1 knockdown trigeminal ganglion sections (Figure 11E,G, arrowheads). Taken together, these findings reveal, at the cellular level, decreased condensation of undifferentiated neural crest cells and placode‐derived neurons within the forming trigeminal ganglion.

### 2.6 Deficits in neuronal projections are apparent at later developmental timepoints after Elp1 knockdown

**FIGURE 12:**
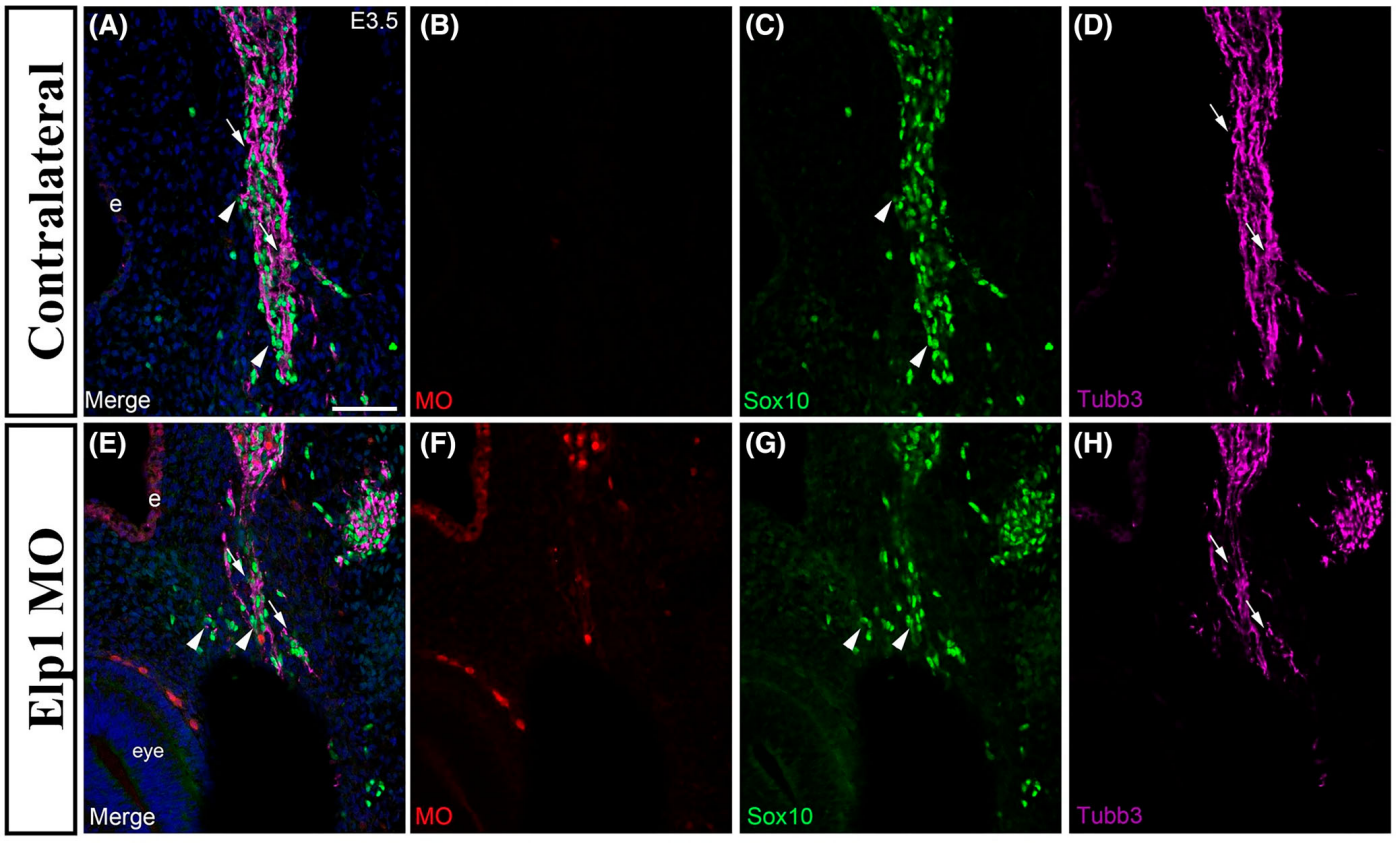
Axon outgrowth appears reduced after Elp1 knockdown. Representative transverse section through the forming ophthalmic branch of the trigeminal ganglion of an Elp1 MO‐treated embryo at E3.5/HH20, with the contralateral control (A–D) and Elp1 MO‐treated (E–H) sides shown. Immunohistochemistry for Sox10 (A,C,E,G, green, labels neural crest cells) and Tubb3 (A,D,E, H, purple, labels placode‐derived neurons). MO‐positive cells are visualized by the lissamine tag on the MO, which fluoresces red (F; none in B), with corresponding merged images of all channels with DAPI (A,E, blue, marks all nuclei). Arrowheads point to Sox10‐positive neural crest cells (A,C,E,G), while arrows indicate Tubb3‐positive neurons (A,D,E,H). Scale bar in (A) is 50 μm and applies to all images. e = ectoderm.

Later in trigeminal ganglion development (E3.5/HH19‐20), undifferentiated neural crest cells and placode‐derived neurons are tightly intermixed, with neurons extending axons to target tissues. In this stage group, the ophthalmic projections are easily distinguishable in cross‐sections through the trigeminal ganglion (Figure 12A,E). The projections from the contralateral control side trigeminal ganglion exhibited well‐organized Tubb3‐positive axons (Figure 12A,D, arrow), with Sox10‐positive neural crest cells lining the axons as expected (Figure 12A,C, arrowhead). After Elp1 knockdown (Figure 12E–H), we noted an apparent reduction in axon extension to peripheral targets, identified by Tubb3 immunoreactivity (Figure 12E,H, arrow) compared to unelectroporated, contralateral control trigeminal ganglia. However, the distribution of Sox10‐positive neural crest cells along or adjacent to the neuronal projections was still maintained (Figure 12E,G, arrowhead). These results further support a role for Elp1 in controlling proper outgrowth of axons from placode‐derived neurons during trigeminal ganglion development.

## 3 DISCUSSION

Development of the cranial sensory ganglia is a very complex and highly coordinated process. For example, neural crest cells and placode‐derived neurons must work together through reciprocal interactions to form the trigeminal ganglion,^3^, ^5^, ^6^, ^7^ which is involved in the perception of many sensations in the head and face, including pain, touch, and temperature.^1^ While the cellular origin of the trigeminal ganglion is firmly established, the molecules involved in trigeminal ganglion development are still not known. To fill this knowledge gap, we identified Elp1 as a candidate for regulating chick trigeminal gangliogenesis. Intriguingly, mutations in *ELP1* cause FD, a fatal disorder that broadly affects the nervous system.^27^, ^28^, ^29^ Symptoms associated with FD, along with prior studies in mouse models, point to trigeminal nerve developmental defects.^11^, ^12^, ^13^, ^30^ While the expression and function of Elp1 in neural crest‐derived trigeminal neurons has been characterized in a mouse model of FD,^16^ the role of Elp1 in trigeminal placode cells had yet to be studied. To this end, we used the chick embryo to examine Elp1 expression and function in trigeminal placode cells and subsequent trigeminal ganglion development. The results of these experiments showed that knockdown of Elp1 in trigeminal placode cells leads to disruption of trigeminal ganglion development in specific ways.

### 3.1 Elp1 is expressed in the proper spatio‐temporal pattern to function in trigeminal ganglion development

While our data revealed ubiquitous Elp1 expression in the chick embryo at the stages we evaluated (E1.5‐3.5/HH11‐20), we observed enrichment in the ectoderm, including where the trigeminal placodes will form; migratory neural crest cells; and undifferentiated neural crest cells and the cell bodies, but not axons, of placode‐derived neurons within the trigeminal ganglion (Figures 2, 3, 4, 5). Section immunohistochemistry identified a punctate cellular distribution of Elp1 throughout these timepoints. Although evidence in the literature supports the presence of only placode‐derived neurons at these stages,^3^, ^24^, ^25^, ^31^, ^32^, ^33^, ^34^, ^35^, ^36^, ^37^, ^38^, ^39^, ^40^, ^41^ only one of these studies involved rigorous fate mapping, but up to E2.75/HH18.^41^ We acknowledge that additional fate mapping studies of the chick trigeminal ganglion are needed to confirm the current literature and clarify the timing of appearance of placode‐ and neural crest‐derived neurons.

Interestingly, previous studies in the chick trunk showed that Elp1 was not expressed in migratory neural crest cells but was detected in the cell bodies and axons of neural crest‐derived dorsal root ganglion neurons.^20^ A later study also reported Elp1 expression only in post‐mitotic neurons of the dorsal root ganglia.^19^ These immunohistochemistry data, however, were acquired using an Elp1 antibody that is no longer commercially available, with a different epitope sequence than the antibody used in our studies. Therefore, resulting expression patterns may be due to epitope location and/or antibody affinities. In addition to the antibodies used, differences between trunk and cranial development are relatively common^24^ and may explain the expression pattern and distribution for Elp1 we observed in our current studies.

In the E10.5 mouse trigeminal ganglion, Elp1 is enriched in the cell bodies and axons of neurons, with little to no Elp1 present in undifferentiated neural crest cells and a general lack of nuclear staining observed.^16^ While these immunohistochemical results are consistent with the expression pattern we noted in the forming chick trigeminal ganglion, differences could be attributed to the stages examined. At E10.5 in the mouse, neural crest cells are poised to begin differentiating into trigeminal neurons, beginning at E11.^42^ The latest stages examined in our chick studies, however, are still at least a day before the onset of neural crest cell differentiation into neurons. Importantly, Elp1 expression has yet to be examined at earlier developmental timepoints in the mouse when neural crest cells begin their migration. However, given these collective findings and conservation between chick and mouse development, we would expect to observe axonal expression of Elp1 in trigeminal placode‐derived neurons at later developmental stages. Lastly, our chick data revealed punctate expression of Elp1 in the cell types in which Elp1 was expressed. Given the known localization of Elp1 to intracellular vesicles,^21^ immunohistochemistry with additional antibodies to label these vesicles will aid in identifying the subcellular location of Elp1.

Due to its ubiquitous expression and role in the Elongator complex, Elp1 function specifically in placodal neurons of the trigeminal ganglion can be considered more critically in the context of human disease. Mutations in subunits of the Elongator complex give rise to diseases and conditions with distinct phenotypes, including FD^28^, ^29^, ^43^ (*ELP1*), intellectual disability^44^, ^45^ (*ELP2*), amyotrophic lateral sclerosis^46^ (*ELP3*), and Rolandic epilepsy^47^ (*ELP4*).^9^ These findings support the notion of both Elongator‐dependent and ‐independent roles for Elp subunits. Interestingly, a common theme underlying these conditions is nervous system dysfunction, which gives insight into the importance of Elp proteins specifically in these tissues versus others. This is exemplified by FD, in which a point mutation in the splice site of intron 20 of *ELP1* causes *ELP1* mis‐splicing and consequently the removal of exon 20. This mutation is not completely penetrant, with mis‐splicing occurring most frequently in nervous system tissues but still allowing for the production of some “wildtype” Elp1 transcripts and protein.^30^ However, other non‐neuronal tissues are less affected by this mutation and produce more “wildtype” Elp1 transcripts and proteins compared to nervous system tissues. This observation suggests that splicing and processing of *ELP1* varies among different cell/tissue types and that perhaps sufficient ELP1 protein is present to carry out its functions in these non‐neuronal tissues. Although FD phenotypes are consistent with gross nervous system deficits, with other cell types seemingly unaffected, this latter finding still requires more rigorous testing.

In our studies in the mouse^16^ and herein, we find Elp1 to function in different aspects of trigeminal neurodevelopment. Deletion of Elp1 from neural crest cells does not affect early assembly of the trigeminal ganglion but leads to target tissue innervation deficits, including loss of TrkA neurons through aberrant apoptosis. Conversely, our findings revealed a smaller trigeminal ganglion after knockdown of Elp1 in chick trigeminal placode cells, followed later by a reduction in the innervation of the eye by trigeminal axons. Altogether, our results point to the importance of having sufficient Elp1 protein levels, and ultimately function, in placodal neurons to mediate proper trigeminal ganglion development.

### 3.2 Elp1 knockdown in placode cells negatively impacts trigeminal ganglion development

To evaluate Elp1 function, we used a combination of splice‐ and translation‐blocking MO to deplete Elp1 from trigeminal placode cells. Knockdown was verified through immunoblotting (Figure 6), which revealed decreases in five bands that were Elp1‐immunoreactive except for a band at 150 kDa (assumed to be a background band since its levels did not change). Multiple bands at different molecular weights could point to various Elp1 isoforms, differential modification of Elp1, and/or proteolytic processing of Elp1 that occurs throughout embryonic development, particularly since immunoblotting was performed on tissue dissected from pooled embryos at E3.5 (HH19‐20), which encompasses two embryonic stages. However, our knockdown studies provide a distinct advantage, as they allow us to evaluate Elp1 function in the absence of embryo lethality, which occurs when Elp1 is knocked out in the mouse.^48^ Notably, FD patients do not have a complete loss of ELP1 protein; rather, ELP1 protein levels are only reduced.^30^ Thus, our knockdown system may serve as a more accurate model of FD. Given the phenotypes we observed after this degree of Elp1 knockdown, it is likely that even greater levels of knockdown would cause failure of trigeminal ganglion formation or even be embryonic lethal.

Using these MO, we found a 20% reduction in the area occupied by the trigeminal ganglion during initial formation at E2.5/HH15‐17 (Figure 7). Examination of sections through the trigeminal ganglion at this stage revealed that although both placode‐derived neurons and neural crest cells appeared less condensed, they still engaged in normal reciprocal interactions, with neural crest cells forming “corridors” and surrounding placodal neurons as expected (Figure 11).^31^ Effects like these at early stages of trigeminal ganglion assembly have been previously observed after knockdown of gene expression in one trigeminal ganglion precursor cell type, leading to defects associated with the other precursor cell type.^7^, ^49^, ^50^, ^51^ A day later in development (E3.5/HH19‐20), the 20% decrease in area occupied by the trigeminal ganglion and its nerve branches persisted (Figure 9). However, this reduction is far greater (53%) when evaluating only the eye innervation area (Figure 10). Therefore, it is likely that neurite and/or axon branching is affected by Elp1 depletion from placode cells. In support of this, neurite and/or axon outgrowth deficits are also observed in chick dorsal root ganglion neurons after Elp1 knockdown in neural crest cells^19^ and in our mouse trigeminal ganglion studies.^16^

Interestingly, we noted a more substantial ventral axonal innervation defect in the eye (Figure 10). This could be due to a combination of factors, including the mosaic nature of the electroporation, the presence of different guidance molecules within ophthalmic target tissues, and/or the expression of distinct receptors on ophthalmic axons. With respect to the latter, studies performed in the chick discovered the importance of Semaphorin3A–Neuropilin1 (expressed by trigeminal sensory neurons) signaling during cornea sensory innervation.^52^ As such, it is possible that Elp1 knockdown affects the correct expression, levels, and/or distribution of receptors like Neuropilin1 as axons grow to their targets. Abnormal outgrowth visualized in section further supports defects in axonal development, but undifferentiated neural crest cells were still observed lining Tubb3‐positive axons of placodal neurons (Figure 12), providing additional evidence that neural crest cells are not affected per se after Elp1 knockdown in placodal neurons. Future studies evaluating neural crest neuronal and glial derivatives may, however, shed light on whether any non‐cell autonomous effects are observed at later developmental stages.

While our data revealed that knockdown of Elp1 in chick trigeminal placode cells reduced the size of the trigeminal ganglion at stages prior to neural crest cell differentiation (Figures 7 and 9), this is in contrast to findings in the mouse using a conditional knockout approach in which Elp1 is deleted from neural crest cells.^16^ However, subsequent development in the mouse showed disorganization and straying of trigeminal sensory axons with defects in axonal pathfinding and target innervation, also noted to some extent in our data. While some differences could be due to species variability, it is likely that many are due to direct effects on placode‐derived neurons instead of neural crest cells. Noting earlier phenotypic changes is logical because perturbations are occurring to the first population of trigeminal sensory neurons that are present, given that neural crest cells differentiate into neurons later than placode cells.^53^

Additionally, the ophthalmic branch frontal nerve initially extends axons around the eye in the Elp1 conditional knockout mouse (but less than that seen in control littermates), followed by a retraction of these axons.^16^ This demonstrates that at least some axons can initially reach their destinations. Conversely, the ophthalmic medial and lateral nasal nerves fail to form in this mouse, whereas in our data, innervation, albeit reduced, is observed by all these nerves. The unique phenotypes among branches and between the two cell types investigated suggest a potential difference in the origin of these neuronal derivatives in the formation of the different trigeminal ganglion branches. Moreover, previous studies have reported increases or decreases in axon/neurite branching after Elp1 knockdown depending upon context,^13^, ^19^, ^20^, ^54^ which could be due to time points examined or the identity and environment of specific neurons. Later chick developmental stages comparable to these mouse studies will be beneficial to investigate to distinguish possible context‐dependent differences in Elp1 function between neural crest and placode cell derivatives of the trigeminal ganglion.

### 3.3 Potential mechanisms underlying trigeminal ganglion phenotypes observed after Elp1 knockdown in placode cells

The deficits seen in neuron projections upon Elp1 knockdown in chick trigeminal placode cells suggest defects in axon pathfinding and guidance. Receptors for neurotrophins such as Trks are important for stimulation of intracellular signaling cascades associated with growth and survival of neuron populations. Elp1 is reported to modulate TrkA/NGF retrograde signaling by regulating the phosphorylation of TrkA receptors in signaling endosomes.^21^ One hypothesis is that neurons cannot receive NGF (i.e., insufficient TrkA is present to bind NGF), leading to their death. However, findings also support defects prior to NGF binding, including a reduction in TrkA immunoreactivity and neuron number in the Elp1 conditional knockout mouse, suggesting additional explanations for the observed phenotypes.^16^ Moreover, studies in the mouse trigeminal ganglion identified a primarily neural crest origin for TrkA neurons, while TrkB and TrkC neurons arise from placode cells.^16^ If this relationship is conserved, it is likely TrkB and/or TrkC neurons are being affected when Elp1 is depleted from chick trigeminal placode cells. Immunohistochemistry on Elp1 MO‐treated embryonic trigeminal ganglion tissue using antibodies for TrkA, B, and C will address potential conservation of trigeminal ganglion development between aves and mammals. Other mechanisms and pathways that may be altered upon Elp1 reduction, such as those affecting cytoskeletal regulation, cell adhesion, and vesicular trafficking, have been previously reported^18^, ^19^, ^55^ and are consistent with results from our functional studies. Defects in axon outgrowth could arise from cytoskeletal dysfunction and/or interaxonal adhesion. Investigating possible impacted pathways will be helpful to identify mechanisms underlying Elp1‐mediated trigeminal ganglion development.

Overall, our data provide new and important insights into Elp1 function in the proper development of not only placode‐derived neurons but also the trigeminal ganglion as a whole. Reducing Elp1 levels in chick trigeminal placode cells negatively impacted trigeminal ganglion development. In contrast to the loss of Elp1 in the mouse neural crest cell lineage, earlier defects in trigeminal ganglion development were observed, underscoring the known developmental timing differences in the neuronal contributions of each cell population to this ganglion. Our findings are the first to characterize the expression pattern and role of Elp1 in trigeminal placode‐derived neurons. Notably, our studies add to the growing literature regarding the importance of Elp1 during normal trigeminal ganglion development and how abrogation of Elp1 function leads to trigeminal nerve deficits in FD.

## 4 EXPERIMENTAL PROCEDURES

### 4.1 Chicken embryos

Fertilized chicken eggs (*Gallus gallus*) were obtained from the University of Maryland (College Park, MD) and/or outside vendors (e.g., Centurion, GA). The eggs were incubated at 37°C in a humidified incubator to desired developmental timepoints. After incubation, eggs were windowed to gain access to the embryo. India ink (Pelikan) diluted in Ringer's solution (123 mM NaCl, 1.5 mM CaCl_2_, 4.96 mM KCl, 0.81 mM Na_2_HPO_4_, 0.147 mM KH_2_PO_4_, pH 7.4) was injected under the embryo using a 1 mL syringe with an 18‐G needle to provide contrast when viewing the embryo under a dissecting microscope. Staging of embryos was then conducted according to the Hamburger–Hamilton staging guide.^56^

### 4.2 Morpholinos

A 3′ lissamine‐tagged Elp1 translation‐blocking MO (5′–CAGCAGCCGCAGATTCCTCATGG–3′) and Elp1 splice‐blocking MO (5′–CCTGACAGACGCCTCACCGACTG–3′) were designed according to the manufacturer's criteria (GeneTools, LLC). These MOs were mixed and used at a 1:1 ratio (2 mM total concentration). A standard scrambled Control MO (5′–CCTCTTACCTCAGTTACAATTTATA–3′) prepared by the manufacturer served as a control and was used at a concentration of 2 mM.

The specificity of all MO sequences was verified using the NCBI Nucleotide BLAST tool. According to directions from the manufacturer, the inverse complement of the MO sequence was compared with the chicken (*G. gallus*) transcriptome to ensure homology was specific to desired targets, thereby mitigating the possibility of off‐target effects.

### 4.3 *In ovo* ectodermal electroporation

Electroporation of the embryo ectoderm to target forming placode cells was performed as previously described.^26^ Briefly, a 27 1/2‐G needle was used to make a hole above the embryo head, in line with the ectodermal edge, just outside the area opaca. A 1 mm × 0.75 mm 4‐inch glass needle was inserted through the vitelline membrane into the space where the embryo is housed. Elp1 or Control MO was then overlaid on top of the ectoderm at E1.5/HH10‐11 (prior to placode cell delamination) to target forming trigeminal placode cells and their neuronal derivatives. Platinum electrodes (0.5 mm thick) were placed with the positive electrode on top of the chick embryo and the negative electrode beneath. The lissamine tag imparts the MO with a slightly positive charge. With the negative electrode under the embryo, the MO is thus pulled down into the ectodermal cells. Three pulses of 9 V lasting 50 ms with intervals of 200 ms were then applied using an electroporator (California Institute of Technology). Eggs were then re‐sealed using packing tape followed by parafilm and re‐incubated for the desired amount of time depending on the experiment.

### 4.4 Embryo collection, embedding, and sectioning

Embryos were dissected off the yolk at specific stages using the Hamburger and Hamilton staging guide, rinsed in Ringer's solution, and then extra tissue surrounding the embryo was trimmed away. Embryos were fixed in 4% paraformaldehyde (PFA) via submersion and gentle agitation at 4°C overnight (or 45 min at room temperature for wildtype immunohistochemistry). After fixation, embryos were permeabilized and washed 3 times in 1× phosphate‐buffered saline (PBS) with 0.1% Triton X‐100 (PBS‐Tx) for 10 min each. Embryos used for whole‐mount immunohistochemistry were stored in 1× PBS until ready for processing.

All other embryos were put through a sucrose gradient: 5% sucrose (w/v) in 1× PBS at room temperature until embryos sank (~5–10 minutes), followed by 15% sucrose (w/v) in PBS at 4°C overnight. Next, embryos were equilibrated into gelatin by submerging them in 7.5% gelatin (made in 15% sucrose) at 37°C for 8 h and then transitioning into 20% gelatin (made in 1× PBS) at 37°C overnight. After these equilibration steps, embryos were positioned into molds containing 20% gelatin. The gelatin was allowed to solidify and then the entire mold was set on ice for 10 min. Next, the molds containing the embedded embryos were frozen in liquid nitrogen vapor and each embedded embryo was stored at −80°C until further processing. Embryos were sectioned using a cryostat (Leica) at 12–14 μm, and sections were collected on Superfrost Plus charged slides (VWR, 48311–703). Sectioned tissue was used immediately or stored at −20°C until processed for immunohistochemistry.

### 4.5 Immunohistochemistry and tissue clearing

#### 4.5.1 Tissue sections

Slides containing sectioned tissue were either used immediately or removed from storage at −20°C and brought to room temperature. All slides were degelatinized in 1× PBS at 42°C for 20 min. After degelatinization, slides were placed in PBS‐Tx for 30 min. Tissue was permeabilized in 1× PBS/0.5% TritonX‐100 for 5 min and then blocked in 10% heat‐treated sheep serum (HTSS) diluted in PBS‐Tx for 1 h. Primary antibodies were diluted in PBS‐Tx + 5% HTSS as indicated in Table 1 and incubated in a humidified chamber overnight at 4°C. Slides were then washed four times for 30 min each at room temperature in PBS‐Tx to remove any unbound primary antibodies. Next, sections were incubated with secondary antibodies, diluted at 1:500 in PBS‐Tx + 5% HTSS, in a humidified chamber for 1 h at room temperature. Slides were then washed 4 times for 30 min each at room temperature in PBS‐Tx to remove unbound secondary antibodies, followed by 2 rinses in 1× PBS for 5 min each, all at room temperature. Coverslips were mounted with DAPI Fluoromount‐G Mounting Medium (Southern Biotech, 0100‐20) and dried in the dark at room temperature overnight prior to imaging. After drying overnight, slides were stored at 4°C when not being imaged.

**TABLE 1:** Antibodies used for immunohistochemistry (IHC) and immunoblotting (WB).

Antibody	Source	Catalog number	Use/dilution
Elp1 (IKBKAP): mouse monoclonal)	Sigma	WH0008518M3	IHC (1:50)
Tubb3 (β‐tubulin III): mouse monoclonal	Abcam	ab78078	IHC (1:500 for sections; 1:250 for whole‐mount)
Sox10: rabbit polyclonal	GeneTex	GTX128374	IHC (1:500)
E‐cadherin: mouse monoclonal	BD Transduction Laboratories	610182	IHC (1:500)
Elp1 (IKBKAP/IKAP): rabbit polyclonal	LifeSpan BioSciences	LS‐B571	WB (1:10,000)
Beta‐actin: mouse monoclonal	Santa Cruz	sc‐47778	WB (1:10,000)
HRP‐conjugated anti‐rabbit IgG	Rockland	611‐1302	WB (1:15,000)
HRP‐conjugated anti‐mouse IgG1	Jackson Immunoresearch	115‐035‐205	WB (1:15,000)

#### 4.5.2 Whole‐mount

Embryos set aside for whole‐mount immunohistochemistry (stored in 1× PBS at 4°C) were blocked in PBS‐Tx + 10% HTSS for up to 2 h at room temperature with gentle agitation. Embryos were then incubated with primary antibody (Table 1) diluted in PBS‐Tx + 5% HTSS for up to 2 days at 4°C with gentle agitation. Next, embryos were washed 4 times for 30 min each in PBS‐Tx at room temperature, then incubated with secondary antibody diluted at 1:500 in PBS‐Tx + 5% HTSS overnight at 4°C with gentle agitation. After this incubation, embryos were washed 4 times for 30 min each in PBS‐Tx, followed by 2 washes with 1× PBS for 20 min each, all at room temperature. E2.5 (HH15‐17) embryos were imaged at this step, while E3.5 (HH19‐20) embryos were cleared before imaging, as described below.

### 4.6 Fructose and urea solution (FRUIT) clearing

After whole‐mount immunohistochemistry, embryos were cleared using the FRUIT clearing method to better visualize the forming trigeminal ganglion.^57^ Embryos were moved through a series of FRUIT buffers containing 8 M urea (Sigma, U5378), 0.5% (v/v) α‐thioglycerol (Thermo Fisher, T090525G), with increasing concentrations of fructose (Sigma, F3510). Embryos were incubated in 35% FRUIT for 6 h, 40% FRUIT overnight, 60% FRUIT for 8 h, followed by 80% FRUIT overnight. All incubations were performed at room temperature with gentle rocking. Embryos were stored and imaged in 80% FRUIT buffer. Embryos were not stored in 80% FRUIT for more than 2 days prior to imaging due to crystallization occurring if embryos are left for too long in this buffer.

### 4.7 Confocal imaging

All imaging was performed on a Zeiss LSM 800 confocal microscope. Tissue sections were imaged using 5×, 10×, or 20× air objectives, or the 63× oil objective. Embryos processed for whole‐mount immunohistochemistry were imaged in either 1× PBS (Elp1 MO, E2.5 (HH15‐17)) or 80% FRUIT. Z‐stack images were taken at 5 μm intervals using 5× and 10× air objectives. For all applications, laser power, gain, offset, and digital zoom remained the same for Contralateral versus MO‐treated tissue. CZI files were processed using Zen software (Blue edition 2.0, Zeiss) while Z‐stack CZI files were processed in ImageJ using the Z‐project function (Hyperstack mode) to create maximum intensity projections.

### 4.8 Measurements and statistical analysis

Measurements were conducted using the open‐source image processing program FIJI,^58^ which is based on ImageJ software.^59^ Measurements were performed on Tubb3‐labeled maximum intensity Z‐projections described above. The boundary of the trigeminal ganglion, including the nerve projections, was first determined by Tubb3 immunostaining and outlined using the freehand sections tool, followed by measurement of area in the outlined region. Ectodermal Tubb3 staining was cropped out to better visualize the trigeminal ganglion, as it merges with the maxillomandibular branching of the trigeminal ganglion when creating maximum intensity Z‐projections.

Similarly, measurements of the area occupied by axon projections from the ophthalmic branch that innervate the eye were obtained by outlining the nerve innervation starting at the point at which additional branches break away from the main ophthalmic branch. Contralateral and MO‐treated trigeminal ganglia were compared using paired *t*‐tests in Graphpad Prism, with *p* ≤ .05 was considered significant.

### 4.9 Tissue collection

Embryos electroporated with Elp1 MO or the standard Control MO were collected 48 h post‐electroporation. Successfully electroporated trigeminal ganglia were dissected in 1× PBS under a Zeiss SteREO Discovery V8 Pentafluor fluorescent microscope to visualize MO‐positive fluorescent tissue and then pooled for immunoblotting (*n* = 18 Control MO, *n* = 21 Elp1 MO). The tissue was pelleted by centrifuging at 500*g* for 5 min at 4°C and excess buffer was removed, followed by flash‐freezing of tissue in liquid nitrogen, and storage at −80°C until ready for immunoblotting.

### 4.10 Immunoblotting

Tissue pellets were thawed on ice and lysed in lysis buffer (50 mM Tris pH 8.0, 150 mM NaCl, 1% IGEPAL CA‐630) supplemented with 1× complete protease inhibitor cocktail (Roche, 04693124001) and 1 mM PMSF (Roche, 54561623) for 30 min on ice, gently vortexing every 10 min. Samples were then centrifuged at max *g* for 15 min at 4°C and the solubilized protein fraction was collected. Protein concentration was determined using a Bradford assay (Thermo, 1857210) and equivalent amounts of protein per sample were boiled in 1× Laemmli sample buffer at 99°C for 5 min, followed by cooling to room temperature.

Prepared samples were loaded onto a 7.5% sodium dodecyl sulfate–polyacrylamide gel (Biorad, 4561024) and separated by electrophoresis at 70 V for approximately 2.5 h in 1× running buffer (diluted from 10× running buffer [25 mM Tris base, 1.92 M Glycine, 3.5 mM SDS]). To transfer proteins from the gel, 0.2 μm PVDF membrane (Thermo Fisher, 88520) was first equilibrated for 30 s in methanol followed by a 20‐min equilibration in 1× transfer buffer (100 mL 10× running buffer, 200 mL methanol, and 700 mL ddH_2_O). After protein separation, the SDS‐PAGE gel was incubated in 1× transfer buffer for 20 min for equilibration. Proteins were then transferred to PVDF membrane using the Mini Trans‐Blot® Cell (Bio‐Rad) wet transfer system running at 70 V for 2 h at 4°C. Membranes were incubated in a blocking solution (1× PBS + 0.1% Tween‐20 [PTW] + 5% milk) for 45 min at room temperature followed by primary antibody incubation overnight at 4°C, also diluted in blocking solution (Table 1). After primary antibody incubation, membranes were washed in PTW 3 times for 10 min each. Secondary antibodies (Table 1) were diluted in blocking solution for 1 h at room temperature followed by 3 additional washes in PTW for 10 min each. Membranes were incubated with chemiluminescent substrates (Supersignal West Pico PLUS (Thermo Fisher, 34,580) and/or Supersignal West Femto (Thermo Fisher, 34,095)) and developed using a ChemiDoc XRS system (Bio‐Rad). Blots were then stripped using Restore Plus Western Blot Stripping Buffer (Thermo Fisher, 46430) for 15 min, rinsed twice in PTW, and re‐blocked for 45 min in blocking solution. Membranes were then re‐probed using β‐actin (Table 1) as a loading control antibody following the above procedures. Immunoblot analysis was performed using Image Lab software (Bio‐Rad) to determine band size and volume. Relative protein levels were calculated by normalizing to loading control band volumes. Differences in amounts of protein were determined by comparing normalized ratios between Control MO‐ and Elp1 MO‐treated samples, with Control MO‐treated samples set to one.
